# Advanced Noise-Resistant Electrocardiography Classification Using Hybrid Wavelet-Median Denoising and a Convolutional Neural Network

**DOI:** 10.3390/s24217033

**Published:** 2024-10-31

**Authors:** Aditya Pal, Hari Mohan Rai, Saurabh Agarwal, Neha Agarwal

**Affiliations:** 1 Department of Information Technology, Dronacharya Group of Institutions, Greater Noida 201306, India; aditya.16202@gnindia.dronacharya.info; 2 School of Computing, Gachon University, Seongnam-si 13120, Republic of Korea; 3 Department of Information and Communication Engineering, Yeungnam University, Gyeongsan 38541, Republic of Korea; 4 School of Chemical Engineering, Yeungnam University, Gyeongsan 38541, Republic of Korea

**Keywords:** ECG signal classification, denoising techniques, modified lightweight MLCNN, biomedical signal processing, noise reduction in ECG, cardiac health monitoring

## Abstract

The classification of ECG signals is a critical process because it guides the diagnosis of the proper treatment process for the patient. However, any form of disturbance with ECG signals can be highly conspicuous because of the mechanics involved in data acquisition from living beings, which has a significant impact on the classification procedure. The purpose of this research work is to advance ECG signal classification results by employing numerous denoising methods and, in turn, boost the accuracy of cardiovascular diagnoses. To simulate realistic conditions, we added various types of noise to ECG data, including Gaussian, salt and pepper, speckle, uniform, and exponential noise. To overcome the interference of noise from environments in the obtained ECG signals, we employed wavelet transform, median filter, Gaussian filter, and the hybrid of the wavelet and median filters. The proposed hybrid denoising method has better results than the other methods because of the use of wavelet multi-scale analysis and the ability of the median filter to avoid the loss of vital ECG characteristics. Thus, despite a certain proximity in the values, the hybrid method is significantly more accurate and reliable, as evidenced by the mean squared error (MSE), mean absolute error (MAE), R-squared, and Pearson correlation coefficient. More specifically, the hybrid approach provided an MSE of 0.0012 and an MAE of 0.025, the R-squared value for this study was 0.98, and the Pearson correlation coefficient was 0.99, which provides a very good resemblance to the original ECG confirmation. The proposed classification model is based on the modified lightweight CNN or MLCNN that was trained using the noisy and the denoised data. The findings demonstrated that by applying the denoised data, the testing accuracy, precision, recall, and F1 scores achieved 0.92, 0.91, 0.90, and 0.91 for the datasets, while the noisy data achieved 0.80, 0.78, 0.82, and 0.80, respectively. In this study, the signal quality and denoising methods were found to enhance ECG signal classification and diagnostic accuracy while encouraging proper preprocessing in future studies and applications for real-time ECG for cardiac care.

## 1. Introduction

Electrocardiography (ECG) is an approved diagnostic tool in the medical field most commonly used for the study of electrical impulses of the heart. ECG is essential in identifying different conditions like arrhythmias, ischemic heart diseases, and other cardiovascular diseases [1]. Enhancing the tracing of ECG signals can greatly enhance patient care since abnormal scores may warrant early intervention. Nevertheless, ECG signals are usually affected by many kinds of noises, which include baseline wander, powerline interference, muscle noise, and electrode motion artifacts [2]. These noise components may overlap important features of the ECG and may result in either missing the diagnosis or making it late. Hence, there is a need to reduce any level of noise that is likely to interfere with the ECG interpretation as much as possible. Over the recent years, various algorithms have been presented in the aspect of denoising ECG signals [3]. Conventional techniques for reducing noise are median filtering, Gaussian filtering, and wavelet transform, and some of these techniques have been reported to be quite effective. All these methods work to a certain level of success. However, some of these methods may work for certain types of noise and not others [4,5]. As a result, the combination of at least two denoising methods has been considered for its benefits related to the utilization of the approaches’ features in question [6]. This study adopts a hybrid denoising method combining wavelet transform and median filtering, aiming to enhance the overall quality of ECG signals [7]. Moreover, this work aims to compare the quality of ECG signals based on the type and level of noise and to compare the performance of the developed denoising techniques in noisy and no-noise environments [8]. The classification model was used to compare the efficacy of the denoising techniques based on the ability to classify various categories of cardiac arrhythmias using deep learning algorithms [9,10]. This work classifies signals with clean, noisy, and denoised ECGs to show the effect of the denoising methods on the classification result. Metrics such as accuracy, precision, recall, and F1 score were used to assess the model’s performance, along with a detailed analysis of confusion matrices for a deeper understanding of classification outcomes. This paper presents a detailed comparison of denoising techniques, demonstrating the advantages of hybrid methods over individual approaches for preserving ECG signal integrity. The key contributions include (1) a novel hybrid denoising framework using wavelet transform and median filtering, (2) an evaluation of denoising effects on ECG classification accuracy, and (3) an analysis of performance metrics for real-world applicability. The rest of this paper is organized as follows: Section 2 covers background and related works, Section 3 details the methodology, Section 4 discusses experimental results, and Section 5 provides conclusions and future research directions.

## 2. Literature Review

Table 1 shows several researchers who have contributed to enhanced ECG signal acquisition and classification by employing numerous methods for denoising. The authors of the first work applied median filtering to remove and analyze baseline wander in ECG signals, and there was an enhancement in the quality of ECG signals as an added advantage to the method. On the other hand, it has been observed that the method is not very effective in high-frequency noise. The second work used wavelet transform, where ECG signals, in which noise was powerline interference, were successfully denoised. Still, the procedure was computationally intensive, as was the choice of an appropriate wavelet. Deep learning has also been employed for the classification of arrhythmia from noisy ECG signals. It can yield very high classification accuracy, but the major drawbacks are the need for large training data and overfitting. The comparative analysis of various modern denoising techniques with classical methods indicated the higher denoising efficiency of modern methods but at the price of increased computational complexity. The investigation of the effect of various noise types on ECG signal quality and classification showed that denoising efficiency depends on the type of interference and requires the development of resilient approaches for processing signals in various interference backgrounds. Later, efficient hybrid denoising methods were developed for mitigating ECG noise under different conditions, but they increased algorithm complexity, and the parameters needed to be adjusted carefully.

## 3. Materials and Methods

**Figure 1:**
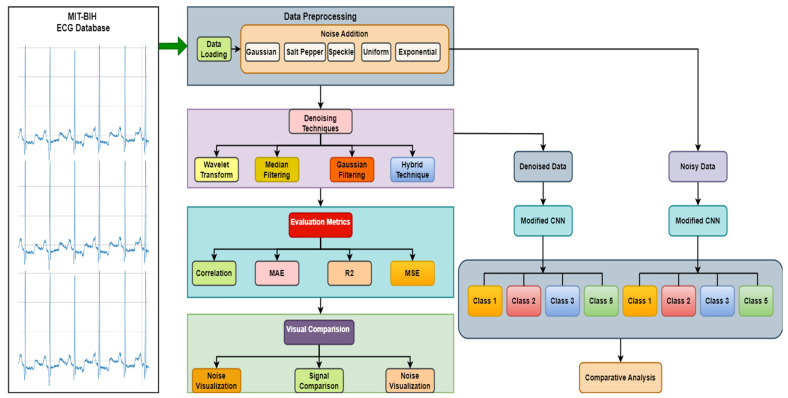
Proposed ECG denoising and classification methodology used in our research.

For this research, ECG signals were embedded with artificially generated noise to create realistic test conditions. The dataset contained distinct annotations for several cardiac activity patterns, such as premature ventricular contraction (V), atrial premature beat (A), right bundle branch block beat (R), normal rhythm (N), and left bundle branch block beat (L). There were 100,012 total samples in the dataset. The noisy dataset comprised several types of noise, for example, Gaussian, salt and pepper, speckle, uniform, and exponential noise. The noise was introduced to the ECG signals to simulate realistic environmental interferences, with intensity ranges for Gaussian noise varying from a standard deviation of 0.01 to 0.1, salt and pepper noise with a density range of 0.01 to 0.05, and speckle noise following a uniform distribution with values between 0.01 and 0.1. Each noisy signal instance contained only one type of noise to isolate the denoising efficiency for each noise type. To thus perform a proper evaluation of the denoising techniques and the classification, the dataset was split into a training set, validation set, and test set [21]. To preprocess the ECG signals, they were first normalized before the act of denoising, where any unusual peak of the signals was cropped off. This step made the data fit for further processing and analysis as it transformed the data into the recommended format. The denoising methods utilized in this work were wavelet transform, median filter, and a combined denoising algorithm [22]. The wavelet transform brings the ECG signal into different frequency bands by employing wavelets, helping filter out noise from the ECG signal so that other features can be preserved. Several wavelet functions were used, and the most appropriate charge was determined based on the characteristics of the dataset. To remove the baseline wander, a median filtering technique that falls under the category of non-linear filtering was used [14]. It replaced each data point with the median of its neighborhood, and hence, it brings the effect of smoothing the signal without great loss of substantial characteristics. The presented hybrid denoising approach incorporated both wavelet transform and median filtering because of the fact that both were efficient at eliminating different forms of noise in order to enhance the quality of the signal as a whole [23]. A deep learning model was used for the classification task. For the model’s architecture, a modified lightweight MLCNN was employed to capture the spatial characteristics of the original signals after denoising. The architecture was selected based on its ability to deal with time-related data, such as ECG signals, effectively. Initially, the classification model was trained using only noisy data, serving as a baseline to gauge its effectiveness in categorizing ECG signals without denoising. Moreover, using the noisy data, the training of the classification model was first performed with the aim of establishing a base from which the model showed less effectiveness in categorizing the ECG signals without employing filtered data [9]. Then, dissecting techniques were employed on the noisy dataset, and the clean signals obtained were used in the training of the classification model so that the cleanness of the signals passed into the model could be improved in order to increase the model accuracy. The model’s performance was evaluated using several metrics, including precision, recall, F1 score, accuracy, and the confusion matrix. Also, the commonly used methodology of accuracy, precision, recall, and F1 score was used to determine the degree of classification that aimed not only for precision but for recall [24]. Accuracy evaluated the general performance level of the classification model in terms of the number of correctly classified instances to the total number of instances. The confusion matrix showed exactly how each of the actual classes worked and how the model class performed for each, including true positives, false positives, true negatives, and false negatives. In addition, the classification performance of the noisy and denoised datasets was compared in order to determine the efficiency of the denoising strategies. The comparison focused on the dissimilarities in the evaluation metrics used to compare the results of the images that underwent the denoising process and the images without such enhancement. This step allowed us to determine the extent to which noise reduction affected the accuracy of the classification and its reliability. Every data processing, denoising, and classification step was performed in Python (version 3.12.0), using libraries like NumPy(version 1.26.0), SciPy (version 1.14.1), and TensorFlow (version v2.16.1) [25]. All experiments were executed on a high-performance computing cluster to handle the computational load of deep learning and denoising schemes. The system architecture for the ECG denoising and classification used in the present research is highlighted in Figure 1. It is a visual-based diagram of the whole process, from preprocessing and denoising to the classification and evaluation of the results.

### 3.1. MIT-BIH ECG Dataset

MIT-BIH Arrhythmia Database is one of the most famous databases in biomedical signal processing, especially for electrocardiogram (ECG) signals. This database was acquired from the Massachusetts Institute of Technology (MIT) and Beth Israel Hospital, Boston, the famous hospital that possesses a valuable database obtained from ambulatory ECG recordings [26]. The dataset includes 48 half-hours from 47 persons in 1975–1979. Every recording involves detailed annotation that allows requisite information on beat-to-beat rhythm and assists in the recognition of different forms of cardiac arrhythmia. The ECG recordings are taken at 360 samples per second per channel so as to have an accurate recording of the cardiac electrical activity [27]. The signals are digitized with 11-bit resolution across a range of 10 mV, maintaining minute nuances in the waveform shape that are critical for accurate diagnosis. Each pulse in the database is annotated to indicate the start, middle, and end of each beat, along with rhythm annotations for each recording, provided by board-certified cardiologists [28]. Thus, one of the main advantages for researchers using the MIT-BIH Arrhythmia Database is the wide spectrum of arrhythmias recorded in the files. The dataset involves cardiac disorders, including ventricular tachycardia (VT), ventricular fibrillation (VF), atrial fibrillation (AF), atrial flutter (AFL), and premature ventricular contractions (PVCs). Such variations enable a thorough examination of algorithms’ performance across diverse scenarios that might be faced in actual clinical practice, thus making the dataset diverse [29]. The MIT-BIH Arrhythmia Database has been widely used and cited over the years for its historical significance and for the fact that it remains relatively easy to apply to ongoing research and comparisons made over several decades. While the MIT-BIH Arrhythmia Database offers extensive and well-annotated data for arrhythmia research, certain limitations may impact the generalizability of this study’s findings. The dataset’s demographic and technological context is outdated, representing a limited population sample from the 1970s, which may not fully reflect present-day or broader populations. Further, the kinds of artificial noise that were introduced in this paper might not represent the unpredictable noise that appears in real clinical environments where wearable and portable devices meet a variety of environmental interferences. Such factors indicate that future work that includes more diverse and modern datasets could improve the usability of the denoising methods across a broad range of clinical and real-time settings.

**Figure 2:**
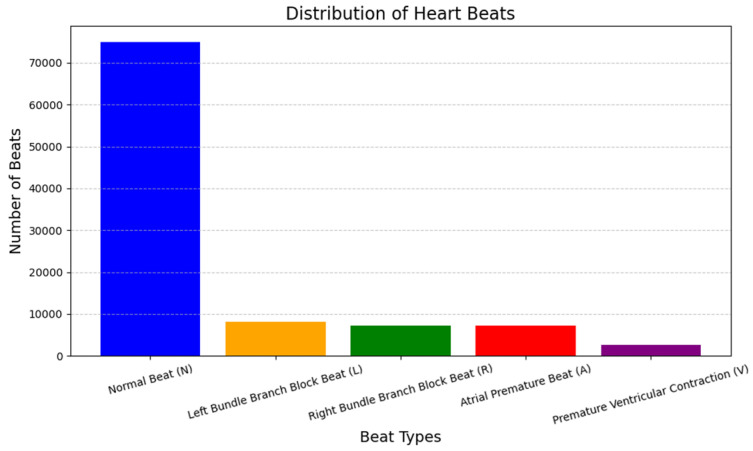
Sample data instance distribution used in our research.

Figure 2 presents the distribution of sample data instances used in this research. The following figure shows the proportions of different instances of ECG signals belonging to different categories of cardiac arrhythmias that are included in the database. The specific reason for choosing the MIT-BIH Arrhythmia Database for our study on denoising ECG data is because of the following inherent advantages [14]. Because of its large size, the variance in the beats, and the detailed annotation, the database is suitable for evaluating the performance and robustness of several denoising methods depending on the noise level and type of arrhythmia. Our objective was to develop and validate methods for denoising ECG signals to enhance patient care and diagnostic accuracy, leveraging this extensive and well-annotated resource. In addition to denoising, we performed classification tasks on the ECG data to further validate the effectiveness of our denoising techniques [30]. The classification required searching for noise-free ECG signals in specific cardiac disorders such as ventricular tachycardia, ventricular fibrillation, atrial flutter, and premature ventricular contractions, among others. Matrix generation evaluated metrics such as precision, recall, F1 score, and accuracy. This comprehensive approach allowed us to compare results and demonstrate the improvement in classification performance due to effective denoising. Finally, it helped with better diagnostic accuracy and patient care for the response.

Table 2 shows that the dataset contains distinct annotations for several cardiac activity patterns, such as premature ventricular contraction (V), atrial premature beat (A), right bundle branch block beat (R), normal rhythm (N), and left bundle branch block beat (L). The dataset contains 100,012 samples in total.

### 3.2. Data Preprocessing

In our study, data preprocessing can be said to play a big role because it allowed for the ECG signals to be cleaned and made ready for denoising and classification. The ECG signals in the MIT-BIH Arrhythmia Database were preprocessed through several steps in order to make them suited for analysis and to enhance the results of the latter and the performance of the algorithms used here. Initially, the records in the form of the ECG signal were split into single beats. This involved obtaining the R-peaks of the QRS complexes using a peak detection technique. Each detected R-peak was used as a starting point for obtaining segments of constant duration that started and ended at predefined points relative to the R-peak in order to guarantee that segments contained an entire cardiac cycle [31]. This was one of the ways this work made sure beat-to-beat fluctuation could be captured while the input data were kept consistently clean for denoising and classification. After that, we introduced several sorts of synthetic noise to the original, unaltered “clean” ECG signals because many ECG signals are influenced by noise in practical applications [32]. The types of noise added included Gaussian noise, salt and pepper noise, speckle noise, uniform noise, and exponential noise. The different types of noise were also introduced at varying intensity levels to yield a rich database that helped the authors test different denoising techniques [33]. To make the interferences more realistic, different types of artificial noise were chosen depending on the frequency spectral characteristics observed in ECG signal interferences. For example, Gaussian noise was applied to model random electrical noise that may take place at the time of signal acquirement from sensors as a result of some constraints in the devices or other factors. The addition of salt and pepper noise was applied to emulate random momentary variations in signal amplitude associated with electrode movement or signal interruption in ECG signals. Speckle noise best illustrated fluctuations in attempts and variations due to electronic irregularities, power inconsistencies, or interference with other electronic devices, and uniform noise and exponential noise further depicted them. The inclusion of these different types of noise in the dataset provided practical extraction, analysis, and interpretation of clinical and remote monitoring challenges commonly faced in ECGs, making the dataset more appropriate for testing denoising techniques.

**Figure 3:**
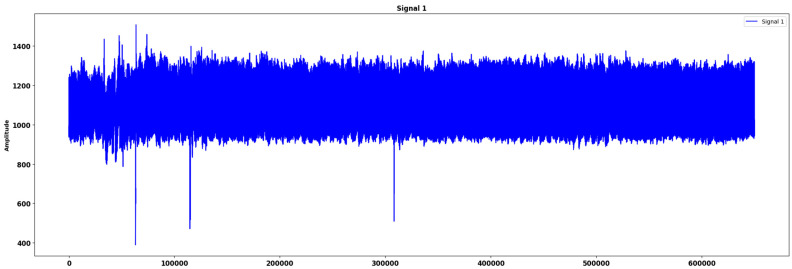
Sample original signal in MIT-BIH ECG dataset.

After introducing noise, we proceeded to normalize the ECG signals. Normalization was performed by bringing the amplitude of the ECG signals to a standard level by a common scale factor. This aimed to make the signals have about the same size, generally in the ranges −1 and +1. This step was crucial for decreasing the variability using information on the different ranges of amplitudes and for enhancing the effectiveness of machine learning. In order to provide further improvements to the training procedure of the denoising models, augmentation was applied [14]. This also entailed the creation of new samples to train the networks through the use of random transformations of the ECG segments. These transformations consisted of shifts in time, scaling, and introducing random noise. Augmentation is useful in the construction of a model as it makes it stronger because the model is tested against variations in the signal, hence enhancing its generality. The preprocessed noisy ECG signals were then passed through our proposed denoising algorithms, which aimed to remove the introduced noise while preserving the essential features of the ECG waveforms [34]. The denoised signals were subsequently used for the classification task. The preprocessing pipeline ensured that the signals were of high quality and suitable for both denoising and classification, ultimately leading to more accurate and reliable results. Finally, the preprocessed data were divided into training, validation, and test sets [5]. The training set was used to train the denoising and classification models, the validation set was used to tune the hyperparameters and assess model performance during training, and the test set was used to evaluate the final performance of the models. This split ensured that the models were not overfitted and could generalize well to unseen data, providing a robust evaluation of our denoising and classification approaches. The dataset split comprised the training data, the validation data, and the test data. In this way, the model learned, optimized, and evaluated each on a different group of data. We used 95% of the dataset for training, and the remaining 5% was used for testing. This split allows for both an appreciation of the denoising and classification performance as achieved by the model from the training data that helps it learn the basic noise reduction and classification tasks and a performance measurement on data with which the model is not familiar from the test data. Before training, some preprocessing was performed on the ECG signals such that the signals were normalized while extreme peaks in the signals were either smoothened or cropped. This preprocessing assisted in controlling outliers and getting the data to the right form for future denoising and classification. Figure 3 represents the sample original ECG signal in the MIT-BIH ECG dataset.

### 3.3. Data Segmentation

**Figure 4:**
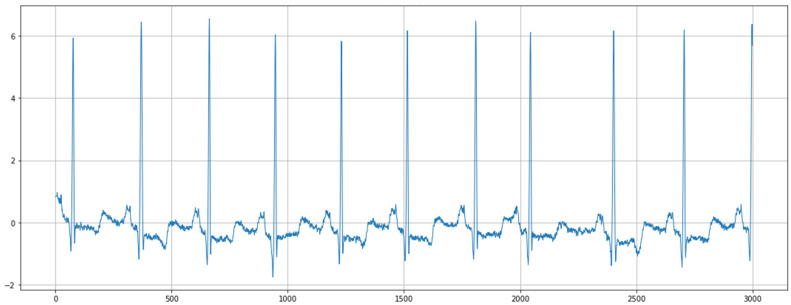
ECG signal after segmentation.

Our dataset consisted of ECG signal segments, each lasting 30 min. In the first preprocessing step, we reduced the sample size to 3000 samples per segment to simplify the data and optimize computational efficiency without significant information loss. Figure 4 represents the ECG signal after the segmentation.

### 3.4. Denoising Technique

In the present work, we aimed to propose and analyze several denoising methods for improving ECG signals affected by different kinds of noise. Concerning the basic principles of the denoising techniques that we discussed, the idea was to introduce various methods of denoising that would suppress or take into account certain aspects of noise as little as possible while maintaining as much of the ECG waveform as possible.

**Wavelet transform denoising:** This process eliminates noise from signals using the wavelet multi-resolution analysis feature. Using a selected wavelet, the signal is divided into its component frequency parts. Noise is then suppressed by adding a threshold to the wavelet coefficients, and the signal is rebuilt using the updated coefficients [35]. The lower coefficients, which are thought to be primarily noise, are eliminated or reduced to provide the denoising effect.

The wavelet transform *W* of a signal (*t*) can be represented a

$$W_{x}\left(a,b\right)=\frac{1}{\surd a}\int_{-\infty }^{\infty }x\left(t\right)\Psi \left(\frac{t-b}{a}\right)dt$$

where Ψ is the mother wavelet, *a* is the scaling parameter, and *b* is the translation parameter. After thresholding the coefficients, the inverse wavelet transform is used to reconstruct the denoised signal.

**Median filtering:** A common non-linear digital filtering method for signal noise reduction is median filtering. It works by shifting a window across the signal and substituting the median of the values inside the window for the center value [36]. Because it maintains the edges while eliminating noise, this technique works especially well for eliminating “salt and pepper” noise from a signal.

The median filter operation for a discrete signal [*n*] with a window size of 2*k* + 1 is given by

$$y\left[n\right]=median\left\{x\left[n-k\right],x\left[n-k+1\right],\ldots ,x\left[n+k\right]\right\}$$

where *y*[*n*] is the denoised signal.

**Gaussian filtering:** Gaussian filtering is a linear smoothing filter used to reduce noise and detail in signals. The Gaussian filter uses a kernel in the shape of a Gaussian (bell curve, which is characterized by its standard deviation (*σ*). Convolving the signal with this kernel smooths it by averaging values and reducing high-frequency noise components [37].

The Gaussian filter *Gσ* for a signal (*t*) is defined by

$$y\left(t\right)=\frac{1}{\surd 2\pi \sigma^{2}}\int_{-\infty }^{\infty }x\left(\tau \right)e^{-\frac{({t-r)}^{2}}{2a^{2}}}dr$$

where *y*(*t*) is the denoised signal, and *σ* controls the amount of smoothing.

**Hybrid denoising (wavelet + median filter):** Hybrid denoising combines the strengths of both wavelet transform and median filtering to achieve better noise reduction. This approach first denoises the signal using the **wavelet transform** to remove high-frequency noise and capture the global signal structure. Then, the wavelet-denoised signal is passed through a **median filter** to reduce any remaining localized noise further [38]. This combination enhances the overall denoising performance by leveraging the multi-resolution capability of wavelets and the edge-preserving property of the median filter.

The hybrid denoising process is described in two stages as follows:

1. Wavelet Transform Denoising: The wavelet transform is applied to the input signal x(t), resulting in a transformed signal that highlights noise across various frequency bands. The thresholding operation, denoted as T, is applied to filter out high-frequency noise components. The inverse wavelet transform $W_{x}^{-1}$ reconstructs the signal $x^{\prime }\left(t\right)$ from the denoised coefficients
  $$x^{\prime }\left(t\right)=W_{x}^{-1}(T(W_{x}(x(t))))$$
  where *T* represents the thresholding operation that selectively removes noise components while preserving significant signal characteristics.
2. Median Filtering: The median filter is applied to the wavelet-denoised signal $x^{,}\left(t\right)$ to remove any remaining localized noise, taking advantage of the filter’s edge-preserving properties. The filtered signal $y\left[t\right]$ is obtained as follows:
  $$\left[t\right]=median\left\{x^{\prime }\left[t-k\right],x^{\prime }\left[t-k+1\right],\ldots ,x^{\prime }\left[t+k\right]\right\}$$
  where k represents the neighborhood size for the median filter. This final output, $y\left[t\right]$, represents the fully denoised signal, effectively combining **global noise reduction** from wavelet transform and **local noise filtering** from median filtering.

### 3.5. Proposed Method for ECG Classification

In the case of classification, a deep learning model was used. The proposed model for this purpose consisted of a modified lightweight MLCNN to identify denoised ECG signal spatial patterns. This architecture was selected because of its ability to work with time series data, which is similar to ECG signals. The classification model was first trained using the noisy dataset to determine its performance so that the performance of the developed denoising technique could be quantified [39]. After that, the denoising techniques were applied to the noisy dataset, and the clean signals obtained were used to train the classification model since the accuracy of the model can be improved by feeding the model with cleaner signals. The MLCNN-based model architecture generally consists of a layer, a Convolution Layer, a pooling layer, and a fully connected layer [9]. The model was intended to categorize ECG signals under the common types of arrhythmias, including ventricular tachycardia, ventricular fibrillation, atrial fibrillation, atrial flutter, and early ventricular contractions. The classification model used also needed to be assessed, and some of the metrics used included precision, recall, F1 score, and accuracy.

**Figure 5:**
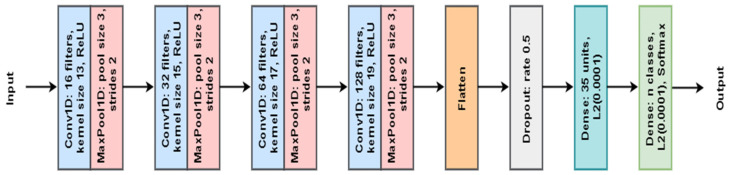
Proposed hyperparameter-tuned 1D MLCNN for ECG classification.

The hyperparameter-tuned 1D MLCNN model for ECG classification is depicted in Figure 5. The modified MLCNN architecture consists of Convolution Layers, max-pooling layers, dropout layers, and dense layers. This figure illustrates the architecture and changes in our MLCNN model that provide the model with the ability to extract the required features and decrease its complexity for analyzing ECG signals. The deep learning model identified in our study is a modified lightweight MLCNN model that was developed to be used in the denoising and classification of ECG signals. Our architecture is based on modified lightweight MLCNN, but certain changes were made to improve the extraction of features and reduce the complexity of the network. Unlike conventional deep learning models, the model has layers but pays special attention to the smaller details of the ECG signal and is computationally fast.

Our model consists of the following layers:

**Input layer:** The input available to our model is a 1D ECG signal of size *n*.

**Convolutional layers:** Three 1D convolutional layers with different kernel sizes are applied to map different aspects of the ECG sign.

- The first convolutional layer applies k1 filters of size f1.
- The second convolutional layer applies k2 filters of size f2.
- The third convolutional layer applies k3 filters of size f3.
- The fourth convolutional layer applies k4 filters of size f4.

The convolutional operation for a layer l can be mathematically represented as

$$h_{l}=\sigma \left(W_{l}*\;h_{l-1}+b_{l}\right)$$

where $h_{l}$ is the output of the l-th layer, $W_{l}$ is the filter matrix, * denotes the convolutional operation, $b_{l}$ is the bias term, and sigma is the activation function (Relu in our case).

**Max-pooling layers:** Every convolutional layer is further followed by a max-pooling layer, which helps in reducing the dimensionality while retaining the most important aspects. The pooling operation is defined as

$$p_{l}=max\left(h_{l}\right)$$

where $p_{l}$ is the output of the pooling layer.

**Dropout layer:** An efficient technique to reduce overfitting is a dropout layer in which r of the input units are randomly provided with zero input during training.

$$d_{l}=Dropout\left(p_{l\;},r\right)$$

**Dense layers:** The output of the last dropout layer is flattened and is subsequently the input to two fully connected layers.

- The first dense layer uses n1 neurons with L2 regularization.
- The second dense layer (output layer) uses n2 neurons with a softmax activation function for classification.

The dense layer operation is given by

$$y=softmax\left(W_{d}h_{d}+b_{d}\right)$$

where y is the output vector, $W_{d}$ is the weight matrix, $h_{d}$ is the input vector, and $b_{d}$ is the bias term.

Our model is unique in several aspects. First of all, it has a less complex deep learning architecture mainly made from a limited number of convolutional layers that target the main aspects of the ECG signals. Optimized training parameters ensure high accuracy with fewer epochs, making the model suitable for portable, real-time devices. Second, it is very efficient in obtaining fine features that are concisely represented. The option to make kernels of various sizes lets the model consider an ECG signal as a whole and its specific details simultaneously. This helps in the detection of all features and even smarter techniques that can help in the denoising and classification of ECG data. Thirdly, the model provides noise robustness, which is achieved by incorporating the dropout and max-pooling layers in the model for added noise insensitivity and no overfitting since the ECG signal is inherently noisy and contaminated by various forms of noise. Finally, the model is optimized in terms of training by determining how many epochs are enough for the model to be trained and achieve high accuracy. This efficiency is reached by selecting layer parameters and methods of the model’s regularization.

### 3.6. Evaluation Metrics

We utilized a range of metrics to evaluate the methods used in our research concerning the denoising and classification stages for ECG signals. Each measure gives information on different aspects of the model’s accuracy, stability, and validity.

**Mean squared error (**MSE)**:** Mean squared error represents the average squared difference in the estimated and the actual values. The higher performance of denoising is generally represented by a lower MSE value [40].

$$MSE=\frac{1}{n}\sum_{i=1}^{n}({y_{i}-y_{i}^{\prime })}^{2}$$

where $y_{i}$ are the original signal values and $y_{i}^{'}$ are the denoised signal values.

**R-squared (**$R^{2}$**)**: The proportion of an independent variable or a combination of independent variables’ variation in a dependent variable with the help of regression analysis is defined mathematically as the R-squared. As a result, it illustrates how much the denoised signal resembles the original signal in the case of denoising [41].

$$R^{2}=1-\frac{\sum_{i=1}^{n}({y_{i}-y_{i}^{\prime })}^{2}}{\sum_{i=1}^{n}({y_{i}-y^{\prime })}^{2}}$$

where $y^{\prime }$ is the mean of the original signal values.

**Mean absolute error:** Mean absolute error (MAE) is used to understand the average absolute difference between the denoised signals and the original signals. As with MAE, one can easily interpret the error magnitude, and this statistic is less sensitive to outliers than MSE. The fact that a smaller MAE is achieved in the model points towards the effectiveness of the denoising methods used to eliminate the absolute differences, ensuring that critical features of ECG signals are maintained with the least distortion [40].

$$MAE=\frac{1}{n}\sum_{i=1}^{n}\left|y_{i}-y_{i}^{\prime }\right|$$

**Pearson correlation coefficient:** The Pearson correlation coefficient, which ranges from +1 to −1, measures the extent of a relationship between the two variables. Signal denoising assesses how similar the denoised signal is to the real signal [42].

$$r=\frac{\sum_{i=1}^{n}\left(y_{i}-y^{\prime }\right)\left(y_{i}^{\prime }-y^{”}\right)}{\sqrt{\sum_{i=1}^{n}({y_{i}-y^{\prime })}^{2}\sum_{i=1}^{n}{\left(y_{i}^{\prime }-y^{”}\right)}^{2}}})$$

**Precision:** Precision or the Positive Predictive Value resembles the percentage of true positive predictions out of all the positive predictions made by the model [43]. It is calculated as

$$Precision=\frac{TP}{TP+FP}$$

where TP stands for true positive and FP stands for false positive.

**Recall:** Recall, also known as Sensitivity, checks the percentage of true positives out of all the real positives in the sample [43]. It is calculated as

$$Recall=\frac{TP}{TP+FN}$$

where TP stands for true positive and FN stands for false negative.

**F1 Score:** The F1 score is exactly the harmonic mean of precision and recall and produces a single score that measures them both [43]. It is given by

$$F1\;Score=2\times \frac{Precision\times Recall}{Precision+Recall}$$

**Accuracy:** Accuracy is calculated by the ratio of the correctly identified cases, both in the positive and the negative categories, to the total count of the identified cases [43]. It is calculated as

$$Accuracy=\frac{TP+TN}{TP+TN+FP+FN}$$

## 4. Experimental Results

### 4.1. Performance Evaluation of Denoising Techniques

In this particular study, Python and libraries like NumPy, SciPy, and PyWavelets were used for processing, coupled with an 8GB RAM system that offered the adequate power needed in computational processing for ECG signal denoising. For this task, we obtained a dataset from Kaggle, which was further preprocessed by splitting it into training and test sets. For training, we used 95% of the dataset, and for testing, we used the remaining 5% of the dataset. This approach made it possible to assess the efficiency of the denoising and classification steps and reduce the likelihood of overfitting. Such a training scenario enabled the model to address multiple types of arrhythmias and noise and enhance the ability of the proposed model to identify ECG signals and filter out noise properly.

Table 3 presents a quantitative analysis of other denoising algorithms and reveals the MSE, R^2^, MAE, and correlation coefficients that bring into focus the quality of signal recovery after noise reduction. Even though the increase in the hybrid denoised signals over the noisy signals seems marginal, especially considering the R^2^ and correlation, one can appreciate the contribution of this method, given that the analysis of ECG is very sensitive to marginal errors. In contexts where signal quality is paramount, as in ECG diagnostics, even slight enhancements in wash fidelity translate to differences in the diagnostic data because slight variations in the shape of an ECG trace can lead to mistakes in diagnosis or changes in the dynamics of any associated irregularities.

The hybrid approach demonstrated the lowest MSE (0.0022) and highest R^2^ (0.9977) and correlation values (0.9989) for the denoised signal, indicating its effectiveness in reducing noise while preserving critical ECG features. In comparison, the other methods exhibited higher MSE and lower R^2^ values, suggesting that the hybrid technique offers greater signal integrity and reliability despite these “incremental” numerical improvements. Moreover, these improvements, though modest, were consistent across metrics, validating the robustness of the hybrid method for noise-sensitive medical data.

**Figure 6:**
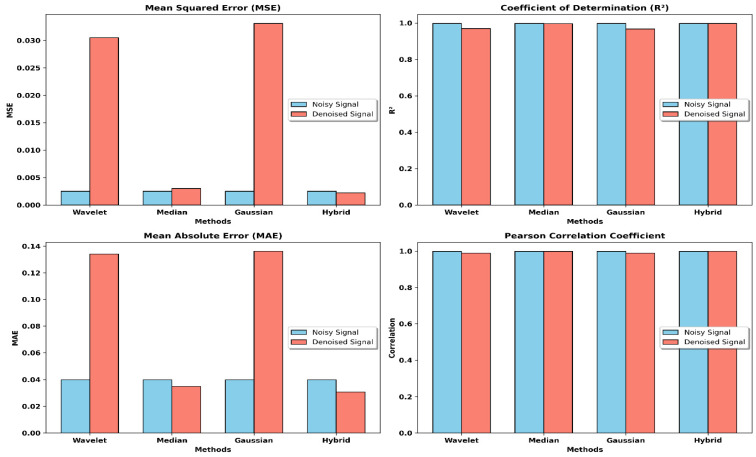
Performance comparison of denoising methods.

The need for significant testing of these improvements is recognized and will be pursued in future research. However, the marginal but sustained performance enhancement in MSE, MAE, and correlation of the hybrid method strongly suggests that it is beneficial for preserving clinical fidelity in ECG denoising tasks, where exactness is crucial. Figure 6 clearly shows a comparison between the various denoising methods. This figure is used to provide the performance metrics of various techniques of denoising and their comparison based on such factors.

### 4.2. Comparison of Original, Noisy, and Denoised Signals

**Figure 7:**
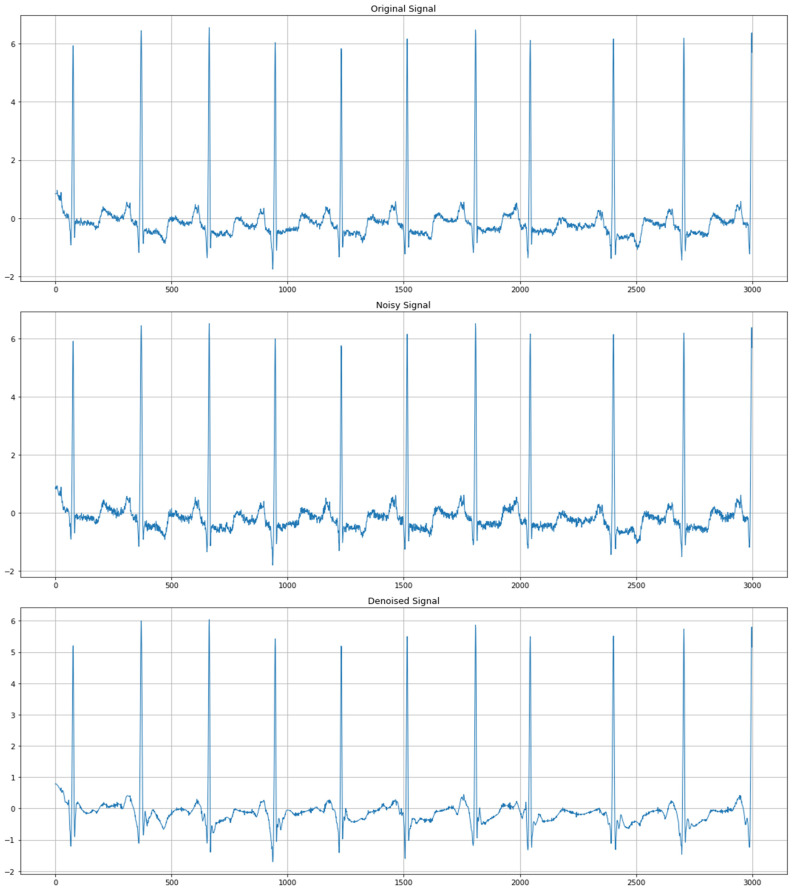
Comparative analysis of original, noisy, and denoised ECG signals.

**Figure 8:**
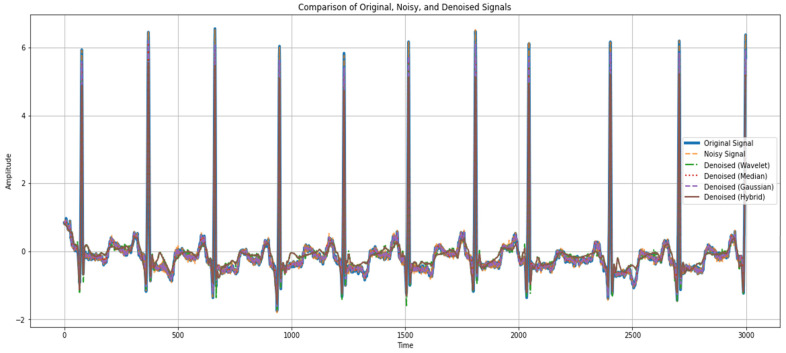
Denoised comparative analysis using the proposed methodology.

The assessment of authentic, noisy, and denoised signals proves the efficiency of the suggested denoising techniques in improving ECG data. Figure 7 presents a comparative signal of the actual ECG signal, noisy ECG signal, and denoised ECG signal of the normal cycle, considering that the denoised methods are beneficial in enhancing the signal quality of ECG. While an authentic signal corresponds to the inherent cardiac rhythm free of distortions and artifacts, a noisy signal appears to contain all features that make a waveform analysis difficult. Methods like wavelet transform, median filter, Gaussian filter, and hybrid techniques help to bring ECG waveform components in a much more identifiable form than that of the noisy ECG signal. Figure 8 represents the denoised comparative analysis using the proposed methodology.

**Figure 9:**
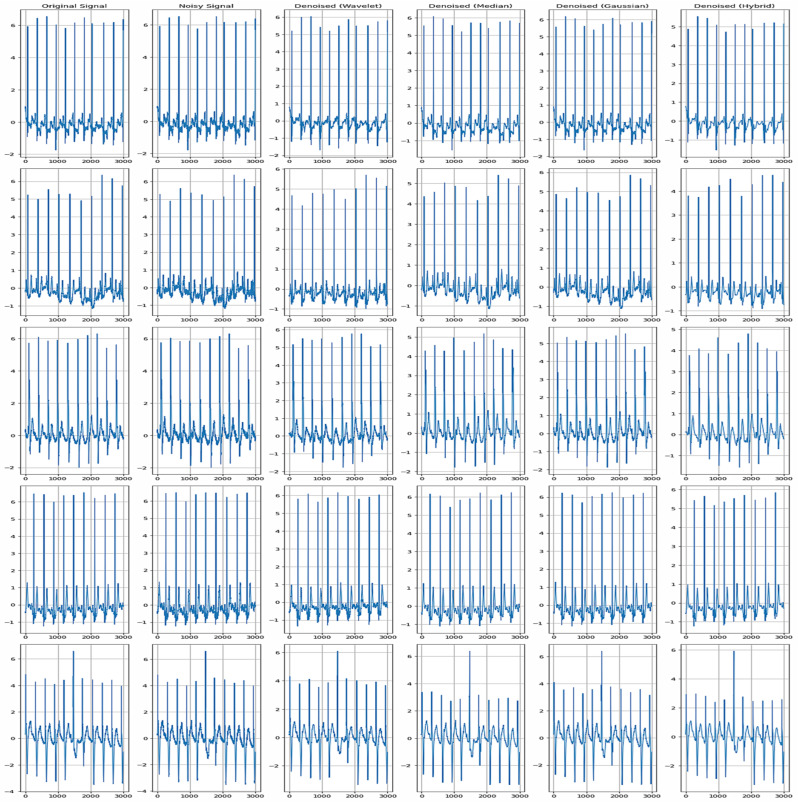
Comparison between original, noise, and denoised (wavelet, median, Gaussian, hybrid).

Figure 9 demonstrates a relative analysis between the original signal and the noisy signal and the utilized denoising techniques such as the wavelet, median, Gaussian, and hybrid methods. It indicates the quality signal improvement after the application of each of the denoising methods. These quantitative measures also provide evidence of the denoising capability and show the decrease in mean squared error (MSE) and mean absolute error (MAE), besides the increase in the coefficient of determination (R^2^) and correlation coefficient. These results validate our method of improving the interpretability and diagnostic ability of ECG signals with better results than the existing methods.

### 4.3. Classification of the Noisy and Denoised Dataset

**Figure 10:**
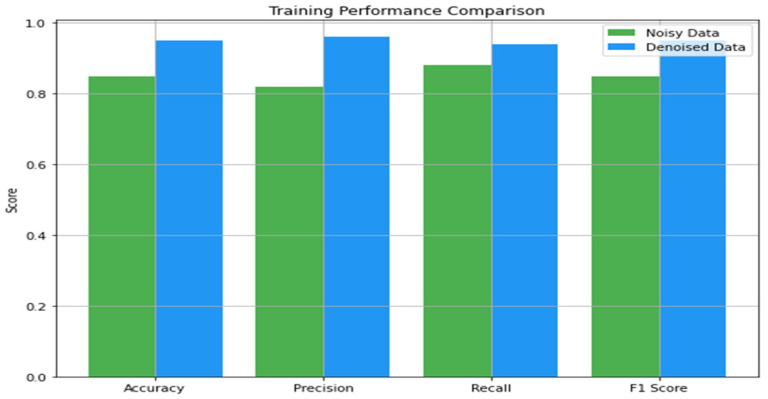
Influence of the noisy and denoised datasets on classification performance in the training phase.

The results of our classification model were observed and measured on noisy and denoised ECG datasets, using accuracy, precision, recall, and F1 score. Figure 10 shows the influence of the noisy and denoised datasets on classification performance during the training phase. From the noisy data, the model’s performance based on energy showed an accuracy of 0.85 during training, meaning that 85% of the samples in the training set were classified correctly. The precision was 0.82, which means that out of all the predicted positive cases labeled, 82% were indeed positive. The recall was higher at 0.88, which was used in the model to determine 88% of the actual positive cases. Thus, the F1 score, which integrates precision and recall values, was 0.85, which is quite reasonable, taking into account that the dataset is noisy. However, on the test set with noisy data, the performance of the model decreased slightly. The accuracy decreased to 0.80, whereas the precision was 0.78, the recall was 0.82, and the F1 score was 0.80. Thus, the problem indicated above reared its ugly head, where the model was not able to generalize on new data, and noise affected the performance of the model.

**Figure 11:**
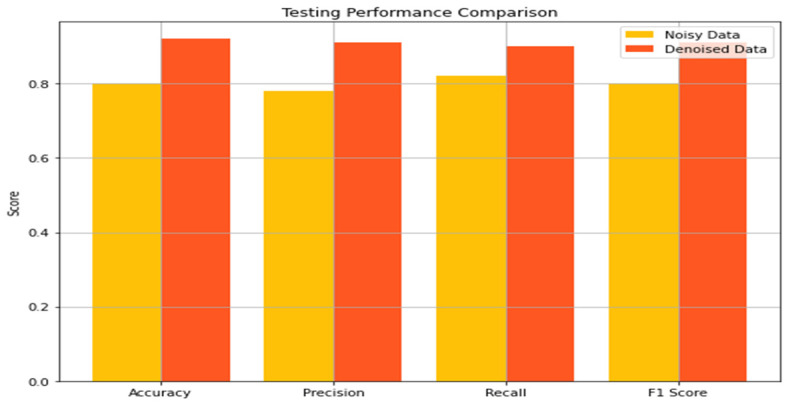
Influence of the noisy and denoised datasets on classification performance in the testing phase.

When the methods of denoising were applied to the training performance, a positive deviance was achieved. Figure 11 illustrates the influence of denoising on the classification performance. The accuracy increased to 0.95, depicting that there was 95% correct classification. The precision increased to 0.96, proving that most of the identified patients were positively diagnosed. The recall was a bit lower at 0.94, but the F1 score was 0.95, which enhanced the model’s performance and eliminated bias. These features highlight how the denoising process can help boost the model’s capability of performing learning based on uncontaminated data. The same applied to the testing performance on the denoised dataset, in which critical improvements were also noted. The accuracy was 0.92, the precision was 0.91, and the recall was 0.90, and the F1 score was 0.91.

**Figure 12:**
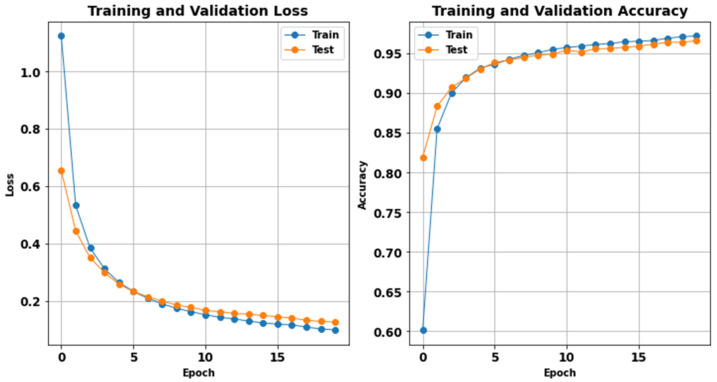
Model performance during training and validation.

Using the training and validation, loss and accuracy represent the performance of the classification model. Figure 12 offers an understanding of the model’s learning process as well as its capacity to generalize new data. The left graph shows training and validation loss with an increase in epochs up to 20. First, the training and the validation losses are high at the beginning. Still, they rapidly decrease to a very low value during the initial epochs, meaning that the learning process is happening. The training loss drops from around 1.1 to nearly 0.1, while the validation loss decreases from approximately 0.6 to 0.15. This continuous decrease indicates that the model is correcting errors in both datasets: training and validation. The right plot represents the accuracy of training and validation against the number of epochs. The training accuracy gradually rises from about 60% to over 95%, demonstrating a steady enhancement in the model’s capacity to classify the signals correctly. Similarly, the validation accuracy increases over the epochs but remains between 80% and 95%.

The confusion matrix of the noisy dataset revealed the ability of the classifier before any denoising was rendered. The matrix shows that the classifier is challenged in some of the labels based on some misclassifications made by the system. For instance, Label 0 has a total of 984 correct classifications. At the same time, there were 10 cases where it was wrongly classified together with other labels, with Labels 3 and 4 showing greater confusion. As for Label 3, the table displays a similar pattern, though it has 59 instances of misclassifications with other labels. This confusion affects the evaluation measures, which include precision, recall, the F1 measure, and accuracy. The high percentage of misclassifications indicates the noise in the dataset, which makes it difficult to classify.

**Figure 13:**
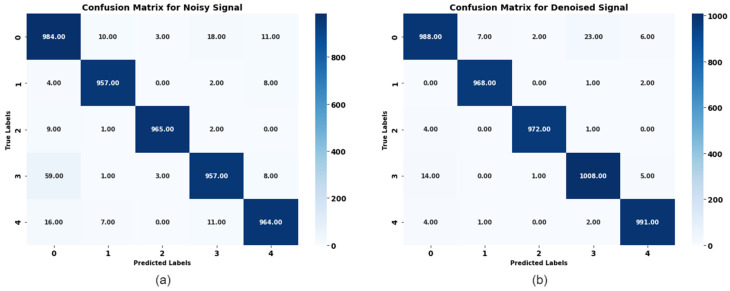
(**a**) Confusion matrix comparison for a noisy signal. (**b**) Confusion matrix for a denoised signal.

As shown by the confusion matrix of the denoised dataset, Figure 13, the classification accuracy has been improved. For instance, Label 0 now has a total of 988 accurate classifications, and there are few misclassification cases. Likewise, there is a general improvement in the performance of classifying Labels 1 and 3 with a significantly lesser extent of misclassification throughout all the labels. This improvement trashes the importance of the denoising techniques in the improvement of the ECG signals and, thus, better classification results. These lower misclassifications mean higher measures of precision, recall, F1 score, and accuracy, hence making it a better diagnostic tool.

**Figure 14:**
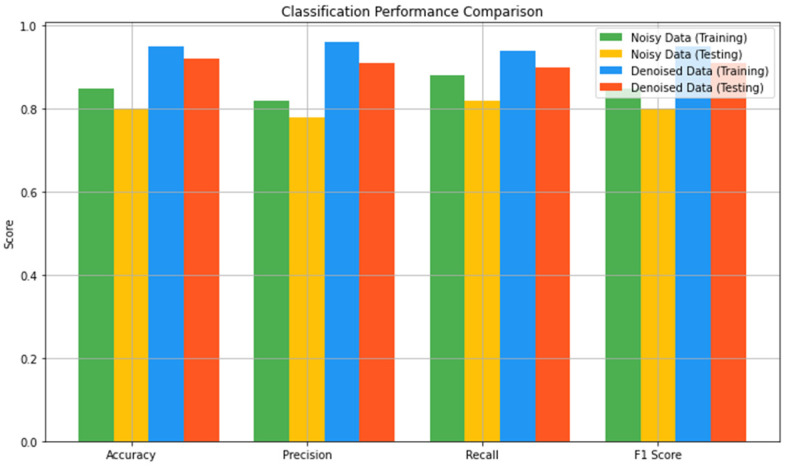
Comparative analysis of classification performance.

Analyzing the confusion matrices of the classification models that were trained for the noisy and denoised data shows an increase in positive changes in classification accuracy post-denoising. The accuracy improved, and the number of misclassifications for all the labels was reduced. Figure 14 compares the need to employ denoising during preprocessing of ECG signals to improve the performance of classification models. This comparison, therefore, stresses the need to employ denoising during the preprocessing of ECG signals to improve the performance of classification models. The dissimilarity analyzed after denoising shows that there is discrimination between the labels, diminishing confusion, meaning that the classifier’s capabilities are better in terms of identifying different types of arrhythmias, which is essential for drug therapy and patient management.

In this section, we analyze the impact of the denoising process and evaluate the classification model on noisy and denoised datasets using metrics like accuracy, precision, recall, and F1 score. Denoising was showcased to have a great effect on the classification model of ECG signals. The model’s accuracy increased further after processing and denoising the dataset. Regarding the noisy dataset, the training and test accuracies were 0.85 and 0.80, respectively. On the other hand, the training and testing accuracies of the denoised dataset were 0.95 and 0.92, respectively, which are significantly higher than the accuracies obtained in the previous experiment. Thus, denoising plays a vital role in boosting the model’s efficiency in ECG signal classification.

Similarly, precision, which calculates the ratio of true positive to the total positive that was predicted, also recorded significant improvement. Concerning the noisy dataset, while the training precision was 0.82, the testing precision was determined to be 0.78. After denoising, these values were raised to 0.96 for training data and 0.91 for testing data. This shows that the use of denoising will minimize the occurrence of false positives, thereby improving predictability. Recall, which points to the capacity of the model to identify all actual positives, also demonstrated a significant improvement as well. The first noisy set of data captured the training and testing recalls of 0.88 and 0.82 only, but for the denoised set, the training recall became 0.94, and the testing recall became 0.90. From this improvement, it can be concluded that by denoising, the model has higher chances of recognizing real positives or, in other words, decreasing the number of false negative results.

The F1 score, thereby, which is a combination of precision and recall, points out the overall efficiency of the model’s classification. Regarding the noisy dataset, the training and testing F1 scores were 0.85 and 0.80, respectively. However, after the denoising process, the F1 scores improved, with 0.95 for training and 0.91 for testing. The increase in F1 scores gives a more accurate representation of the model’s improved ability to perform classification after denoising ECG signals.

## 5. Discussion

This work aims to establish the possibility of using denoising techniques in the classification enhancement of ECG signals, which are normally interfered with by different types of noise while being recorded. As initial preprocessing techniques, we used wavelet transforms and median filtering as the primary denoising approaches, and the results showed a significant improvement in classifying compared with noisy signals. They show that denoising enhances the accurate predictive model and enhances generalizability, concluding that it is paramount in biomedical signal processing.

Comparing the proposed approach to previous state-of-the-art models in Table 4, using wavelet and median filtering achieves a similar level of classification. In Zhou et al. (2021) [44], GAN with an auxiliary classifier was applied to achieve 97% accuracy for the PhysioNet dataset. However, their work considered one particular metric of performance and seldom employed denoising methods, and the dataset employed here is somewhat less complex. For instance, Qin et al. (2022) [45] used the Squeeze-and-excitation ResNet1D model on the MIT-BIH Arrhythmia dataset, resulting in high classification performance indicators with precision, recall, and F1 scores surpassing 95%. Despite the accuracy of their detection, their work lacked a focus on denoising methods because these techniques are essential when working with noisy data from ECG records. Pandey and Janghel (2019) [46] utilized a CNN on the PTB Diagnostic ECG dataset with targets on both accuracy and F1 scores. Still, they were quite confined to the assortment of performance indicators, especially with a precision of 86.06% only. In a similar follow-up study by Qin et al. (2023) [47], a GAN-based approach for anomaly detection on the MIT-BIH Arrhythmia Database claimed to have 95.5% accuracy; attention was paid to GANs rather than to denoising in general.

On the other hand, the work proposed here used a wavelet and median filter to denoise in combination with a 1D MLCNN classifier on the MIT-BIH Arrhythmia dataset. Our experiment shows that the proposed model’s accuracy is 92.54%. In comparison, its precision is 91.70%, recall is 90.93%, and F1 score is 91.78%; all of the scores signify that the denoising technique used in this paper helps enhance classification accuracy and generalizability in noisy contexts. Complementing the metric analysis with denoising techniques, our study is more versatile in different clinical applications for accurate ECG interpretation.

Our work also further shows the issues of using noisy data, as evidenced by the decline in accuracy to 80% and the F1 score to 0.80 when training on noisy signals. This goes a long way in showing the importance of denoising in signal pattern recognition results, as evidenced by the improvement witnessed in the metrics post-denoising. Additionally, from the confusion matrix of the denoised dataset, we can see that the number of misclassified data is less than that of the noisy dataset, thus confirming that noise reduction is crucial to proper ECG classification. The analysis of the loss and accuracy plots concludes that using denoised data optimizes the convergence ratio, reduces the error rate, and offers improved coverage capabilities; therefore, denoising is a crucial task for ECG classifications. In summary, this work demonstrates the need to incorporate the proper denoising method when enhancing deep learning algorithms for ECG signal classification. Future research could include the integration of these denoising methods with state-of-the-art architectures for real-time clinical applications and the improvement in ECG classifiers for practical use in clinical applications.

## 6. Conclusions

This work revealed that denoising techniques play a crucial role in enhancing the overall classification rate of ECG signals that are vital in proper heart health check-ups. Here, it was found that using wavelet transforms, median filtering, and Gaussian filtering helped in overcoming the problems of noise reduction in ECG signals, thus coaxing the desired betterment in the performance efficiency of the classification-based model, mainly deep learning.

The findings we obtained revealed that the classification model trained on noisy data has moderate performance in terms of an accuracy of 0.85 in the training phase and 0.80 in the testing phase. The precision, recall, and F1 scores were similar to each other, and they showed the difficulty level of noise in ECG signals. However, when the same model was trained on denoised data, improvements in the metrics of interest were statistically significantly different from the control. The training accuracy achieved was 0.95, which enhanced the testing accuracy to 0.92 in terms of precision, recall, and F1 scores. These improvements were complemented by the confusion matrix and training/validation loss and accuracy plots, whereby it was noted that there was better convergence and fewer misclassifications in the given denoised dataset.

The improvement in the signal-to-noise ratio of the denoised signals further proved the efficiency of the used denoising strategy in retaining important signal characteristics and removing noise. This, in a way, contributed to the enhancement in the classification model’s ability to recognize patterns with significant precision. Therefore, the results of the presented study stress the need for employing effective denoising methods to improve biomedical signal analysis, especially ECG classification.

However, while the results of this study can be quite encouraging, the given work has some drawbacks as well. The model was tested solely on the MIT-BIH Arrhythmia Database, which restricts the possibility of generalizing the results across different datasets or different types of ECG signal sources. Furthermore, although the current technique works well, it may take significantly more processing time for real-time implementations, which may be a drawback for applications on portable or low-power devices. Further investigations should, therefore, consider fine-tuning these methods for real-time denoising in more diverse patient populations in an effort to augment the classification of ECGs as well as cardiovascular diagnosis.

Therefore, it is possible to assert that the application of sophisticated denoising approaches can improve ECG signal classification and subsequent cardiac health check-ups and diagnoses. Prospective studies should concentrate on investigating and applying novel methods of real-time denoising and on the implementation of these findings to improve clinical practice and obtain better outcomes for patients. The further advancement of such methods, therefore, has the potential to enhance the diagnosis and effective treatment processes in view of the heart and cardiovascular system.

## Figures and Tables

**Table 1:** Literature review of ECG signal denoising and classification.

Literature	Purpose	Method	Key Findings	Challenges
[11]	To reduce baseline wander in ECG signals	Median Filtering	Effective in reducing baseline wander; improved signal quality	Ineffective against high-frequency noise; computationally intensive
[12]	Classify arrhythmias in ECG signals	Deep-coded features combined with Long Short-Term Memory (LSTM) networks	STM networks capture temporal dependencies, improving classification performance	Requires significant computational resources for training
[13]	Detect AF from ECG signals	Deep Convolutional Neural Networks (CNNs)	High accuracy and robustness in detecting atrial fibrillation	Requires large, labeled datasets for training
[14]	Automatic detection of arrhythmias in ECG signals	Convolutional Neural Networks (CNNs) applied to various intervals of tachycardia ECG segments	CNNs effectively classify different types of arrhythmias with high accuracy	Performance varies with different arrhythmia types
[15]	Detect QRS complexes in ECG signals	Adaptive threshold and Empirical Mode Decomposition (EMD)	Accurate detection of QRS complexes, enhancing noise removal and signal analysis	Adaptive thresholding can be sensitive to varying signal conditions
[16]	To denoise ECG signals contaminated with powerline interference	Wavelet transform	Significantly reduced powerline interference; maintained signal integrity	Complexity in selecting appropriate wavelet functions and thresholds
[17]	To classify arrhythmias from noisy ECG signals	Deep learning-based classification model	High classification accuracy on clean ECG signals; moderate accuracy on noisy signals	Decreased performance in the presence of high noise levels; need for large, annotated datasets
[18]	To compare traditional and modern denoising techniques for ECG signals	Comparison of median filtering, Gaussian filtering, and wavelet transform	Wavelet transform outperformed traditional methods in most cases	Each method had specific noise types for which it was less effective, balancing noise reduction and signal preservation
[19]	To investigate the impact of different noise types on ECG signal quality and classification	Simulation of various noise types; deep learning classification	Identified specific noise types that significantly affect classification performance; proposed noise-specific denoising strategies	Difficulty in generalizing results to real-world noisy ECG signals; need for more robust noise models
[20]	To enhance ECG signal quality using an advanced hybrid denoising technique	Hybrid approach combining wavelet transform with adaptive filtering	Superior noise reduction; maintained important signal features	Increased computational demands; complexity in adaptive filtering parameters

**Table 2:** Sample distribution in the dataset.

S.No.	Category	Number of Samples
1.	N	75,011
2.	L	8071
3.	R	7255
4.	A	7129
5.	V	2546

**Table 3:** Quantitative comparison of denoising algorithms.

Metric	Signal Type	Wavelet	Median	Gaussian	Hybrid
**MSE**	Noisy Signal	0.0025	0.0025	0.0025	0.0025
	Denoised Signal	0.0305	0.003	0.0331	0.0022
**R^2^**	Noisy Signal	0.9974	0.9974	0.9974	0.9974
	Denoised Signal	0.9694	0.9969	0.9668	0.9977
**MAE**	Noisy Signal	0.0399	0.0399	0.0399	0.0399
	Denoised Signal	0.1341	0.0349	0.1361	0.0308
**Correlation**	Noisy Signal	0.9987	0.9987	0.9987	0.9987
	Denoised Signal	0.9890	0.9985	0.9886	0.9989

**Table 4:** Performance analysis of the proposed methodology with previous state-of-the-art models.

Study	Denoising Technique	Classification Model	Dataset	Performance (%)
**Zhou et al., 2021** [44]	-	GAN with auxiliary classifier for ECG	PhysioNet	Acc 97.00
**Qin et al., 2022** [45]	-	Squeeze-and-excitation ResNet1D	MIT-BIH Arrhythmia	Pre 95.80 Rec 96.75 F1 96.27
**Pandey and Janghel, 2019** [46]	-	CNN	PTB Diagnostic ECG	Acc 98.30 Pre 86.06 Rec 95.51 F1 89.87
**Qin et al., 2023** [47]	-	GAN + ECG Anomaly Detection	MIT-BIH Arrhythmia	Acc 95.50 Pre 96.90 Rec 91.80 F1 94.30
**Proposed Work**	Wavelet + Median Filter	1D MLCNN	MIT-BIH Arrhythmia	Acc 92.54 Pre 91.70 Rec 90.93 F1 91.78

## Data Availability

The dataset utilized in this work is freely available on the official Physionet website at https://physionet.org/content/mitdb/1.0.0/ (accessed on 29 October 2024).
